# Investigating the Mechanisms of Hydrogen Embrittlement Resistance in Pre-Strained CoCrNi Medium-Entropy Alloy via Hydrogen Migration and Trapping Behavior

**DOI:** 10.3390/ma19143010

**Published:** 2026-07-13

**Authors:** Zening Wang, Sirui Jing, Yu Yan

**Affiliations:** Beijing Advanced Innovation Center for Materials Genome Engineering, Corrosion and Protection Center, Institute for Advanced Materials and Technology, University of Science and Technology Beijing, Beijing 100083, China; wangzeningning@163.com (Z.W.); jing_sr@163.com (S.J.)

**Keywords:** medium-entropy alloy, hydrogen embrittlement, hydrogen microprinting, hydrogen distribution, EBSD

## Abstract

Hydrogen embrittlement (HE) is a critical issue that constrains the service reliability of structural alloys in hydrogen-rich environments. For the CoCrNi medium-entropy alloy (MEA), the interplay between deformation twins (DTs) and HE remains controversial, and the mechanism by which pre-strain-induced twin boundaries (TBs) influence hydrogen migration pathways and fracture behavior still requires further elucidation. To address this, the present study employed multiple complementary techniques, including slow strain rate tensile (SSRT) testing, electron backscatter diffraction (EBSD) analysis, direct hydrogen visualization via hydrogen microprinting (HMP), and microhardness measurements, to comparatively investigate the hydrogen-induced cracking behavior of the alloy subjected to pre-strain levels of 0%, 30%, and 50%. Experimental results reveal that dense nanoscale TBs can serve as both effective hydrogen trapping sites and diffusion barriers, substantially modifying the hydrogen distribution pattern and preventing substantial hydrogen enrichment at grain boundaries (GBs). This twin-dominated regulatory mechanism significantly suppresses hydrogen-induced intergranular fracture, endowing the material with outstanding HE resistance. These findings elucidate the intrinsic anti-HE mechanism governed by twin structures and provide a microstructural design basis for the development of high-performance hydrogen-resistant multi-principal element alloys (MPEAs).

## 1. Introduction

As a prevalent and destructive failure mode of metals, hydrogen embrittlement (HE) triggers crack nucleation and subcritical crack growth, eventually causing abrupt structural failure and degradation of ductility, toughness and strength [1]. With the rapid development of hydrogen energy and high-performance structural materials, the demand for alloys with both superior mechanical properties and excellent HE resistance has become increasingly urgent. Multi-principal element alloys (MPEAs) are composed of multiple principal elements in near-equiatomic proportions, featuring high atomic disorder and forming a stable solid solution structure with high entropy. Compared with conventional alloys, the CrCoNi medium-entropy alloy (MEA) exhibits superior mechanical properties under extreme conditions, such as high toughness at cryogenic temperatures [2,3], making it a promising candidate for cryogenic hydrogen transport and storage applications, including liquid hydrogen storage tanks [4,5]. In such applications, the issue of HE requires particular attention. Although some MPEAs show great potential for solid-state hydrogen storage, understanding HE is critical to enhancing storage efficiency and durability, as local lattice defects, distortions, and dislocations influence hydrogen storage and release capabilities [6]. Compared with conventional face-centered cubic (FCC) alloys, the CoCrNi MEA exhibits a low stacking fault energy (SFE), which facilitates the formation of deformation twins (DTs) during plastic deformation, thereby achieving a synergistic enhancement of strength and ductility [7,8]. However, a systematic understanding of its HE behavior and the underlying mechanisms of hydrogen-microstructure interactions remains lacking, which severely limits its engineering applications in hydrogen-containing environments.

In recent decades, extensive research on the HE behavior of high/medium entropy alloys has confirmed that hydrogen distribution, transport, and cracking behavior are highly dependent on microstructural features such as grain boundaries (GBs) [9,10], dislocations [10,11], precipitates [12,13], and twin boundaries (TBs) [14]. Among these, DTs play a particularly critical role in HE, yet the findings remain highly controversial. On one hand, deformation twin boundaries (DTBs) can act as effective hydrogen traps, promoting hydrogen enrichment and inducing cracking along TBs [15,16,17]. On the other hand, TBs can impede dislocation motion [18,19], and therefore theoretically suppress dislocation-mediated hydrogen transport, leading to a more uniform hydrogen distribution and reduced hydrogen-induced stress concentration [20]. DTs not only affect hydrogen behavior, but relevant studies have also documented the effects of hydrogen on TBs in MPEAs. Zhou et al. [21] investigated the mechanisms underlying the high HE resistance of FeCoNiCrMn alloys through computational simulations and showed that the presence of hydrogen atoms reduces SFE, promotes DTs, and thereby enhances ductility. Cheng et al. [22] demonstrated that the introduction of hydrogen reduces the SFE of CoCrFeNiMn, promotes the formation of more nanoscale DTs and intersecting twin systems, and thereby generates additional work hardening. Zhu et al. [23] found that in the CoCrFeNi MPEAs, the hydrogen-induced passivation mechanism of coherent TBs significantly enhances the resistance of TBs to dislocation slip transmission, revealing that coherent TBs are not weakened but rather can improve the HE resistance of the material through the passivation effect in hydrogen environments. Collectively, these studies demonstrate a complex and bidirectional interaction between DTs and hydrogen in MPEAs, where twins can either trap hydrogen or impede its transport, while hydrogen in turn modulates twin formation and boundary stability, ultimately governing the overall HE resistance.

Prior studies on the HE resistance of CoCrNi MEA have largely concentrated on the effects of precipitates [24], grain size [25], and alloying elements [20,26]. Yan et al. [25] found that as the hydrogen charging current density increases, the HE susceptibility of CoCrNi MEA decreases, accompanied by the appearance of clear slip bands within the grain interiors. Their results indicate that hydrogen promotes localized slip and DTs, and that hydrogen-enhanced localized plasticity (HELP) along with hydrogen-induced decohesion are the possible mechanisms. K. Soundararajan et al. [27] found that hydrogen plays dual roles in the deformation microstructure of CoCrNi MEA. It promotes dislocation tangling at low strain and induces massive nanotwins at high strain. Hydrogen-induced intergranular cracking serves as a weakening mechanism, whereas hydrogen-facilitated nanotwinning brings about dynamic strain hardening as a strengthening mechanism. The competition between these two mechanisms determines the HE resistance of the alloy. Lu et al. [28] found via computational simulations that hydrogen lowers the vacancy formation energy in CrCoNi MEA and drives the breakup of large vacancy clusters into smaller ones. This size-selective destabilization is attributed to the reduction in unstable SFE by hydrogen. Rhode et al. [29] demonstrated that the release of hydrogen from the corresponding traps in CoCrNi MEA requires high energy, that hydrogen diffusion exhibits a delayed effect, and that a considerable amount of hydrogen remains trapped even at elevated temperatures. Hydrogen primarily occupies weak trapping sites, such as interstitial sites and dislocations [30]. Clearly, current research is primarily concerned with the behavior and role of hydrogen in CoCrNi MEAs, such as hydrogen diffusion, the impact of hydrogen on defect structures, and the effects of hydrogen on microstructure during deformation. Current research mainly revolves around three competing mechanisms: hydrogen segregation at GBs causing intergranular weakening, mobile dislocations transporting hydrogen atoms and exacerbating localized damage, and DTs playing a dual competitive role by both acting as hydrogen traps that induce cracking and impeding dislocation motion to enhance resistance against embrittlement, which leads to contradictory conclusions in the literature. Current microstructural control strategies are largely confined to static approaches such as grain refinement, precipitation strengthening, and alloying, while there is a critical lack of visual experimental evidence tracking hydrogen migration behavior within nanotwin structures introduced by pre-straining, and this gap constitutes the key bottleneck that constrains breakthroughs in mechanistic understanding in this field. For the CoCrNi MEA with low SFE, pre-straining, cold rolling, and low-temperature deformation can introduce a large number of DTs. However, how pre-strain-induced DTs regulate hydrogen transport behavior in the CoCrNi MEA remains a core scientific issue that has yet to be clarified. In particular, the dynamic interactions among dislocations, DTs, and hydrogen during plastic deformation, as well as the fundamental mechanism by which twins enhance the resistance to HE, still lack direct experimental evidence and systematic elucidation.

**Figure 1:**
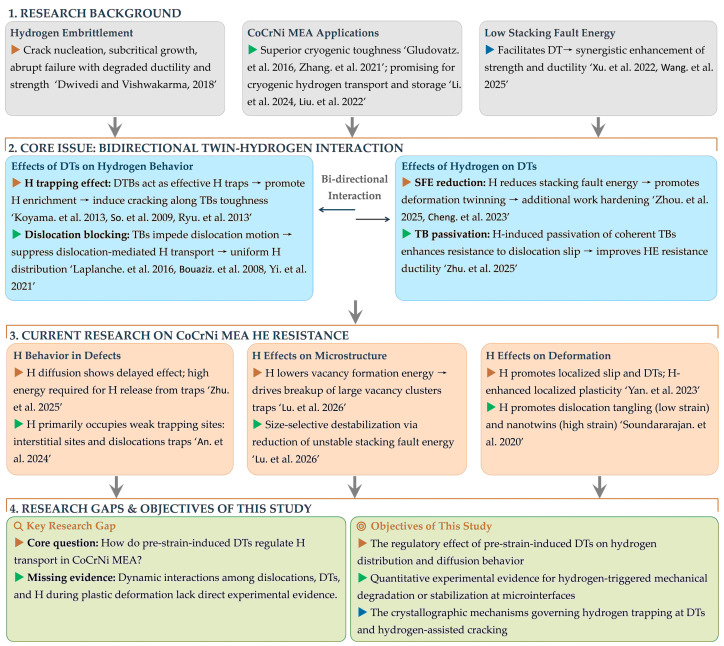
Schematic diagram of current research status on HE behavior of CoCrNi-based MPEAs [1,2,3,4,5,7,8,15,16,17,18,19,20,21,22,23,25,27,28,30].

Our prior study established a macroscale structure–property correlation between pre-strain-induced DTs and enhanced HE resistance via mechanical testing, SEM fractography, and electron backscatter diffraction (EBSD), which confirms the critical role of DTs in suppressing brittle intergranular fracture [31]. Nevertheless, the lack of in situ and quasi-in situ characterization techniques leaves the spatial distribution of hydrogen, the preferential hydrogen diffusion pathways in microstructures, and the fundamental interaction mechanisms between TBs and hydrogen atoms poorly understood. To address these gaps, this study fabricated equiatomic CoCrNi MEAs with 0%, 30%, and 50% pre-strain to obtain microstructures with distinct dislocation and twin densities. All specimens were subjected to electrochemical hydrogen charging and subsequent SSRT tests to evaluate their HE susceptibility. This study adopts two novel characterization approaches: (i) HMP for direct microscale visualization of hydrogen distribution and migration pathways, and (ii) microhardness testing for quantitative evaluation of hydrogen-triggered local mechanical degradation or stabilization inside grains and along GBs. Combined with high-resolution EBSD and geometrically necessary dislocation (GND) density analysis, the above characterizations elucidate three previously unreported mechanisms for CoCrNi MEAs: (1) the regulatory effect of pre-strain-induced DTs on hydrogen distribution and diffusion behavior, (2) quantitative experimental evidence for hydrogen-triggered mechanical degradation or stabilization at microinterfaces, and (3) the crystallographic mechanisms governing hydrogen trapping at TBs and hydrogen-assisted cracking in twinned CoCrNi MEAs. Ultimately, this study establishes a comprehensive physical model that describes the sequential process whereby pre-strain introduces nano-twins, TBs competitively trap hydrogen, hydrogen distribution becomes homogeneous, and GB decohesion and cracking are inhibited. This study provides a self-consistent, experimentally validated theoretical framework for clarifying the fundamental mechanisms by which DTs improve the HE resistance of CoCrNi MEAs. Figure 1 illustrates the current research landscape of CoCrNi-based MPEAs mentioned above, along with the objectives of the present study.

## 2. Materials and Methods

### 2.1. Material Preparation

**Figure 2:**
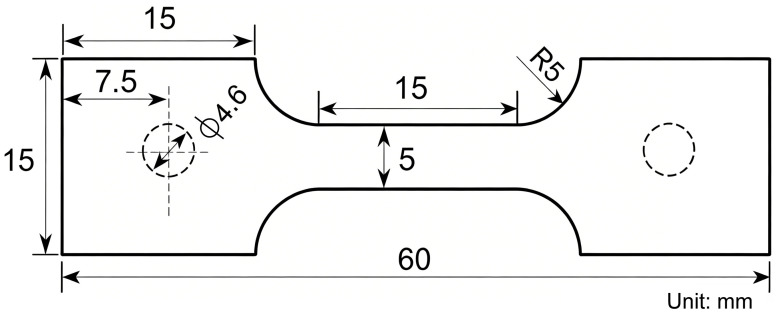
Diagram of the tensile test specimen (the dashed circles indicate that the sample clamping end passes through the mounting hole).

High-purity cobalt, chromium, and nickel (>99.9%) were used as raw materials. A 2 kg equiatomic CoCrNi MEA ingot was fabricated via vacuum levitation melting. After homogenization, the ingot was hot-rolled at 1100 °C with a total thickness reduction of 50% over 8 rolling passes. Multi-pass gradual hot rolling was adopted to eliminate casting segregation, shrinkage cavities and internal metallurgical defects, trigger full dynamic recrystallization, and homogenize elemental distribution throughout the matrix. This was followed by room-temperature cold rolling with a 40% thickness reduction across 6 passes; small single-pass reductions guaranteed uniform plate thickness and prevented edge cracking while accumulating abundant stored dislocations to facilitate controllable DT generation during subsequent tensile pre-straining, yielding a 1.5 mm-thick plate. The plate was then annealed at 1100 °C for 30 min under argon protection and immediately water-quenched to room temperature. This matched annealing-quenching procedure fully removes cold-rolling work hardening and produces homogeneous, fully recrystallized equiaxed grains with intrinsic annealing twins, establishing a consistent baseline microstructure for P0, P30 and P50 specimens where tensile pre-strain is the sole experimental variable. Tensile specimens were machined from the heat-treated plate along the rolling direction, with overall dimensions of 60 mm × 5 mm × 1.5 mm and a gauge section of 15 mm × 5 mm × 1.5 mm, as shown in Figure 2. All specimens were ground stepwise to 5000 grit using SiC paper to ensure consistent surface quality. Pre-straining was performed on a universal testing machine at a constant strain rate of 1 × 10^−3^ s^−1^, with applied engineering strains of 0%, 30%, and 50%. The resulting specimens with distinct microstructures were designated as P0, P30, and P50, respectively [31]. Pre-straining induces cross-sectional reduction in the gauge region. For the P50 specimen, the gauge cross-section decreased from 5 mm × 1.5 mm to 4.2 mm × 1.2 mm. To ensure identical mechanical testing conditions, the gauge cross-sections of P0 and P30 specimens were also machined and polished to 4.2 mm × 1.2 mm, while the gauge length remained 15 mm for all specimens.

### 2.2. Hydrogen Charging and Hydrogen Embrittlement Susceptibility Assessment

Electrochemical hydrogen charging was performed in a solution of 0.5 mol L^−1^ H_2_SO_4_ + 2 g L^−1^ thiourea using a DC power supply, at a current density of 50 mA cm^−2^ for 48 h. Compared with gaseous hydrogen charging, electrochemical charging allows precise control of hydrogen flux and can introduce high hydrogen concentrations in a relatively short time. Slow strain rate tensile tests were conducted at room temperature on an Instron 5967 machine (Instron, Norwood, MA, USA). Tests were run at a constant strain rate of 5 × 10^−5^ s^−1^ until failure. The engineering stress–strain curves were generated using force data recorded by the load cell and crosshead displacement data from the testing machine. Engineering stress was calculated by dividing the applied force by the original cross-sectional area of the gauge section, and engineering strain was derived from the crosshead displacement divided by the original gauge length [32,33]. All specimens were prepared and clamped identically to ensure consistency. This low rate promotes dynamic hydrogen–dislocation interactions, enabling accurate assessment of HE susceptibility, which was quantified by total elongation loss ($EI_{\mathrm{loss}}$), defined as:

(1) $$EI_{loss}=\;\frac{EI_{free\;}-\;EI_{H\;\;}}{EI_{free\;}}\times 100\%$$

where $EI_{\mathrm{free}\;}$ is the tensile elongation of the H-free tensile test specimens, and $EI_{H\;}$ is the tensile elongation of the H-charged tensile test specimens. Hydrogen content was quantitatively measured via thermal desorption spectroscopy (TDS) using a Bruker G4 PHOENIX DH instrument (Bruker Corporation, Ettlingen, Germany). The specimens with dimensions of 15 mm × 1.2 mm × 4.2 mm were heated to 800 °C and held for 15 min to ensure full hydrogen desorption. All TDS measurements were repeated three times to ensure experimental accuracy and data reproducibility.

### 2.3. HMP Characterization

HMP was utilized to visualize and characterize the microscopic hydrogen distribution in the alloy. A uniform photosensitive emulsion was prepared by thoroughly mixing silver bromide (AgBr) powder with sodium nitrite (NaNO_2_) solution under dark conditions. Following electrochemical hydrogen charging, the specimens were rinsed with deionized water, rapidly dried with cold air, and immediately immersed in the emulsion for 2 h at room temperature in darkness. Active hydrogen atoms diffused outward from the matrix and triggered a redox reaction (${\mathrm{Ag}}^{+}\;+{\;H}_{ads}\;\text{→}\;\mathrm{Ag}\;+\;H^{+})$ that reduced silver ions into metallic silver particles, which selectively precipitated at hydrogen-enriched sites. After cleaning, fixing, and drying, the silver particle imprints accurately corresponded to localized hydrogen accumulation, allowing direct identification of hydrogen distribution and enrichment behavior in the alloy microstructure.

### 2.4. EBSD Characterization

All specimens were electrolytically polished prior to EBSD characterization to eliminate surface residual stress and mechanical damage. All EBSD observations after hydrogen charging were performed at the central gauge section of tensile specimens, where full hydrogen saturation is achieved to reveal authentic hydrogen-induced damage in the matrix. Electropolishing was performed in a mixed solution containing 10 vol.% perchloric acid and absolute ethanol at a constant temperature of 273 K under a steady applied voltage of 25 V. This treatment effectively removed surface machining defects and residual stress, producing flat, stress-free, high-quality surfaces for accurate EBSD measurement. After polishing, the specimens were thoroughly cleaned and dried to meet the requirements of subsequent microscopic characterization. The acquired EBSD data were systematically analyzed to identify the crystallographic orientation, GB distribution, and twin structural features, as well as the propagation paths of hydrogen-induced cracks. The corresponding results clarified the inherent relationship between microstructural evolution and hydrogen-induced cracking behavior in the alloy.

### 2.5. Microhardness Testing

Microhardness measurements were conducted using a high-precision digital Vickers hardness tester (Model HVS-1000Z (Shanghai Caikon Optical Instrument Co., Ltd., Shanghai, China), load accuracy ±0.5%). An automated loading-dwell-unloading sequence was programmed with the following parameters: an applied load of 0.01 kg (10 gf, ~98.07 mN), a loading rate of 50 μm s^−1^, a dwell time of 15 s, and a post-unloading stabilization period of 2 s to ensure optimal indentation imaging. To guarantee both data accuracy and statistical significance, systematic indentation arrays were strategically positioned within grain interiors and in the vicinity of GBs. A minimum of five valid indentations was obtained for each distinct microstructural region, with adjacent indentation centers spaced at greater than 2.5 times the diagonal length of the impression to preclude mutual interference from work-hardened zones. The resultant hardness values for each region were computed as the arithmetic mean accompanied by the standard deviation and presented in graphical format.

## 3. Results

### 3.1. Mechanical Properties

**Figure 3:**
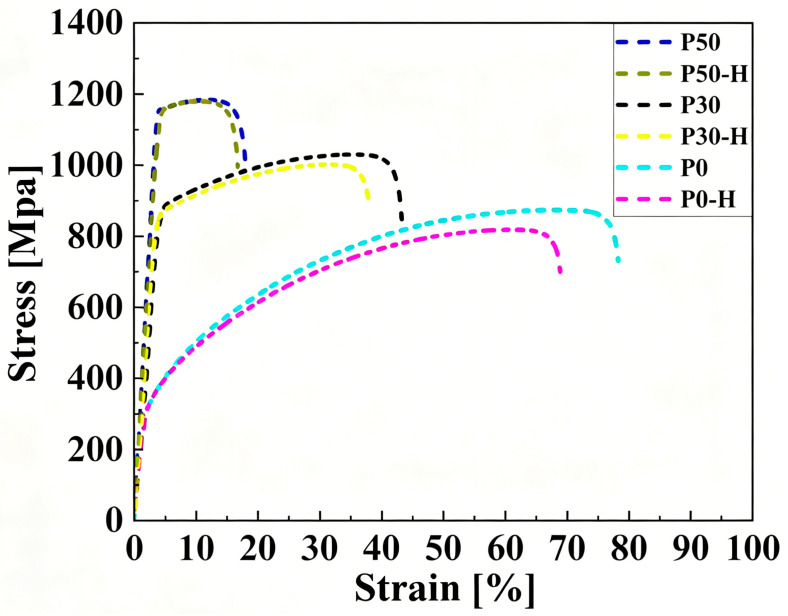
Engineering stress–strain curves of P0, P30, and P50 and their H-charged specimens.

SSRT tests were performed to systematically characterize the mechanical responses of as-received and pre-strained hydrogen-charged specimens. All curves plotted in Figure 3 were averaged from measurements of three replicate specimens to guarantee the reliability and reproducibility of experimental outcomes. Key mechanical parameters were extracted from tensile datasets, including Young’s modulus, yield strength, ultimate tensile strength, strength coefficient, strain-hardening exponent, and fracture stress. Full statistical evaluations covering the minimum, maximum, mean, median, range, and standard deviation were carried out for every mechanical property. Corresponding tensile curves and comprehensive statistical data are available in the Supplementary Information, as presented in Figure S1 and Tables S1–S6. Combined with the prior EBSD microstructural characterization results [31], the P0 specimen exhibited typical annealed microstructural features, achieving the highest ductility (77.62 ± 0.67%) at the expense of a low yield strength (293.79 ± 5.44 MPa). After 30% pre-straining, a dislocation multiplication mechanism dominated the strengthening process, substantially increasing the yield strength of P30 to 882.14 ± 6.24 MPa. However, the work-hardening effect induced by dislocation pile-up concurrently reduced the ductility to 43.49 ± 1.68%. When the pre-strain reached 50%, the interaction between high-density dislocations and nano-scale twins formed a dual strengthening mechanism, further elevating the yield strength of P50 to 1163.88 ± 4.40 MPa. Nevertheless, the obstruction of dislocation motion by TBs, together with the accumulation of geometrically necessary dislocations, significantly exacerbated work hardening, leading to a sharp decrease in elongation to 16.07 ± 0.54%.

Furthermore, the introduction of hydrogen had a negligible effect on the strength of the CoCrNi MEA but significantly deteriorated its ductility. Specifically, comparisons of elongation between uncharged and hydrogen-charged specimens revealed that P0 decreased from 77.62% to 67.79%, P30 decreased from 43.49% to 38.48%, while P50 only marginally decreased from 16.07% to 15.63%. Based on these data, the elongation loss ($EI_{\mathrm{loss}}$) values for the three specimen groups were calculated to be 12.67%, 11.51%, and 2.74%, respectively. It is noteworthy that P0 and P30 exhibited similar degrees of hydrogen-induced ductility degradation, whereas the $EI_{\mathrm{loss}}$ of P50 was significantly reduced, revealing a substantial improvement in its HE resistance. This performance enhancement can be attributed to the introduction of numerous deformation nano-scale twins in the pre-strained microstructure of P50. The TBs act as efficient hydrogen traps, capturing diffusible hydrogen atoms and hindering their enrichment at crack tips, thereby suppressing HELP depletion and crack propagation.

### 3.2. Characterization and Mechanism of Hydrogen-Induced Cracking

Building upon our prior study [31], which established the overall trend of decreasing HE susceptibility with increasing pre-strain, this study selects two extreme microstructural states, the unstrained annealed condition (P0) and the highly pre-strained condition with dense nanoscale DTs (P50), for in-depth comparative characterization. EBSD and HMP techniques are employed to elucidate the specific mechanism by which hydrogen influences mechanical properties after DT introduction, with the aim of revealing the intrinsic correlation between crack initiation sites and crystal structure, thereby advancing the understanding of HE mechanisms in pre-strained CoCrNi MEAs.

**Figure 4:**
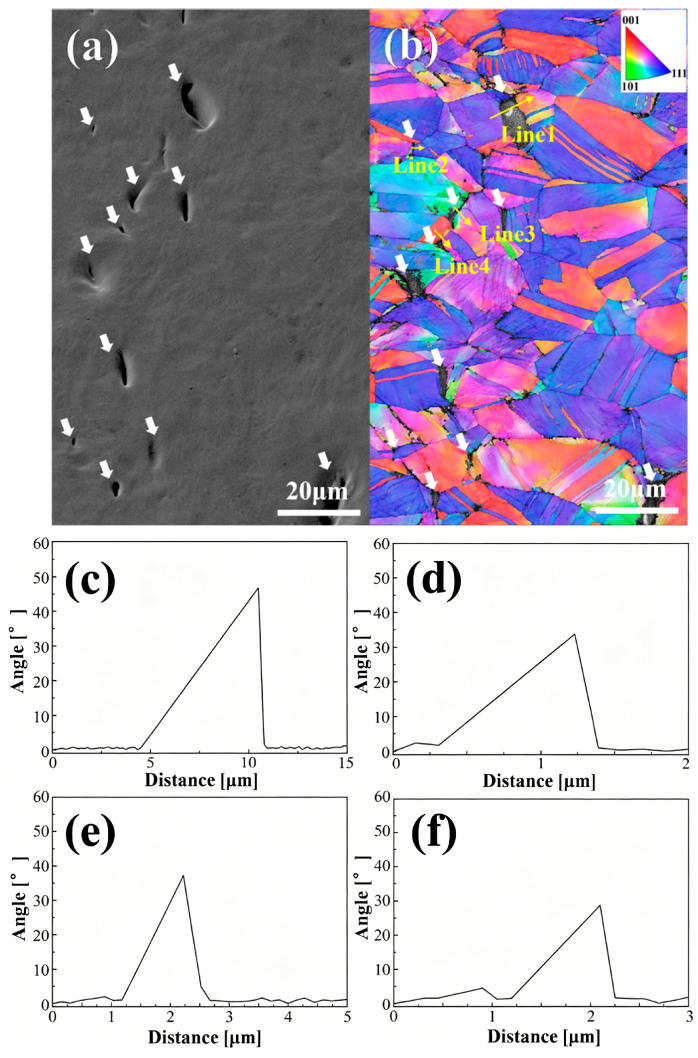
EBSD image of hydrogen induced cracks on the P0 surface: (**a**) SEM image, with white arrows showing the crack location; (**b**) IPF image, where white arrows correspond to crack sites consistent with those in subgraph (**a**), yellow lines lines 1–4 are the paths for misorientation angle measurement; (**c**–**f**) misorientation angle statistics, with the statistical regions corresponding to lines 1–4 in (**b**).

Figure 4a,b present the scanning electron microscope (SEM) morphology and the EBSD inverse pole figure (EBSD-IPF) image of hydrogen-induced cracks in the P0 specimen, respectively. It can be clearly observed from the figures that the cracks exhibit multi-source initiation characteristics without a distinct single main crack, and most cracks originate at GBs. This phenomenon has been widely reported in single-phase FCC structured hydrogen-charged alloys [27,34,35], indicating that GBs serve as synergistic sites for hydrogen segregation and stress concentration, providing the thermodynamic driving force for hydrogen-induced crack nucleation. According to the hydrogen- enhanced decohesion mechanism (HEDE), GBs act as effective hydrogen trap sites, readily capturing and accumulating hydrogen atoms diffused into the material. The accumulated hydrogen atoms weaken the metallic bonding force at GBs, reduce the cohesive strength of the GBs, and thereby induce intergranular cracking. In recent years, further studies have also shown that hydrogen not only weakens GBs but also promotes the formation and aggregation of vacancies near GBs, reducing the local plastic deformation capability and ultimately accelerating crack initiation and propagation [36,37,38].

To gain a deeper understanding of the influence of GB characteristics on hydrogen-induced cracking behavior, quantitative characterization and statistical analysis of the misorientation angles between grains on both sides of the cracks were further performed, with the results presented in Figure 4c–f. The analysis indicates that hydrogen-induced cracks in the P0 specimen predominantly occur at high-angle GBs (HAGBs), while crack initiation is rarely observed at low-angle GBs (LAGBs), exhibiting a significant GB misorientation-dependent preferential cracking characteristic. Previous studies have confirmed that HAGBs (15° < θ < 62.8°) possess a higher degree of atomic misalignment and greater interfacial energy, making them more favorable channels for hydrogen atom aggregation and preferential crack propagation. In contrast, LAGBs (2° < θ < 5°) exhibit a structure closer to that of subgrain boundaries, with lower interfacial free energy that reduces hydrogen atom adsorption and segregation, thereby conferring greater resistance to hydrogen-induced cracking [39,40,41]. This misorientation-dependent hydrogen-induced cracking behavior further demonstrates that GB type is a key microstructural factor affecting the HE susceptibility of single-phase FCC alloys.

**Figure 5:**
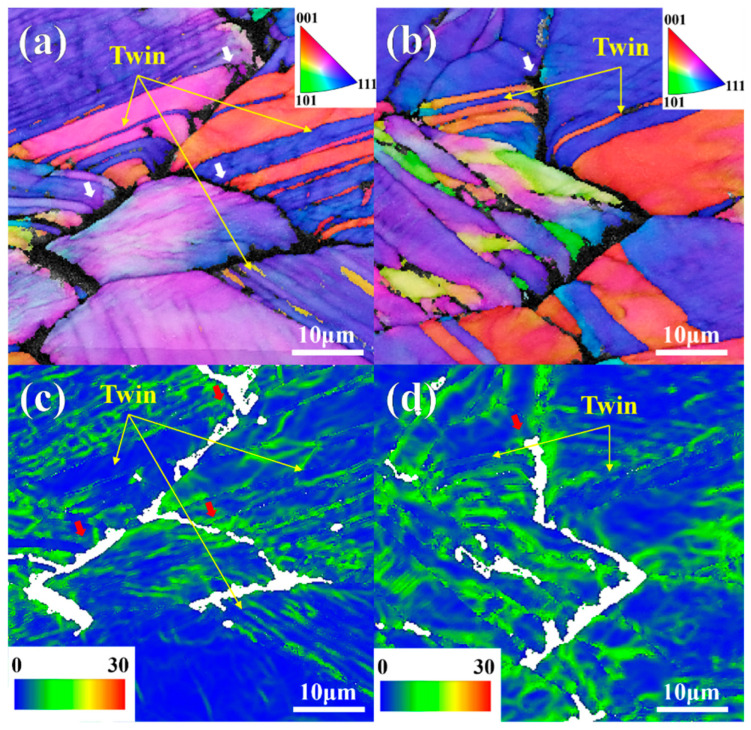
Microstructural and strain analysis of hydrogen-induced cracks in the P0 specimen after hydrogen charging and slow strain rate tensile testing: (**a**,**b**) magnified EBSD images showing hydrogen-induced cracks (white arrows) and DTs (yellow arrows); (**c**,**d**) corresponding GND density distribution maps, where yellow arrows denote deformation twins and red arrows mark regions with high GND density.

To further reveal the initiation mechanism of hydrogen-induced cracks, the microstructural characteristics of crack formation sites in the P0 specimen were systematically characterized, with the corresponding results presented in Figure 5. Figure 5a,b clearly show that cracks predominantly propagate along GBs, exhibiting typical intergranular fracture characteristics. Notably, a substantial number of DT structures were observed within the grains surrounding the crack initiation sites. This phenomenon indicates that DTs play an important role in the hydrogen-induced cracking process. The interaction between DTs and GBs can induce significant local stress concentration, particularly at locations where twins terminate at GBs [42,43]. Such stress concentration not only arises from dislocation pile-up caused by the geometric configuration between twins and GBs but may also be further amplified by the diffusion and aggregation of hydrogen atoms. This mechanism has been experimentally confirmed in studies on the HE behavior of TWIP steel, where the interaction between DTs and GBs, combined with hydrogen-induced local cohesion reduction, significantly increases the likelihood of intergranular crack initiation [15,44].

Furthermore, high-resolution EBSD technology was employed to quantitatively characterize the GND density in the region near the cracks, with the results presented in Figure 5c,d. Red arrows indicate the dislocation-enriched zones along the crack tip and propagation path. The analysis shows that the GND density is significantly higher in the crack initiation zone and near the DTBs compared to other regions. In contrast, within the grains where no DTs have formed, the GND density remains at a relatively low level. This observation is consistent with the statistical trends of GND density for the P0, P30, and P50 pre-strained specimens reported in our previous study [31], namely, that TBs, serving as strong interfacial barriers to dislocation slip, exhibit a continuous increase in density with rising pre-strain levels, which in turn directly drives a significant elevation in the overall intragranular GND density. This established trend provides systematic data support for understanding how pre-strain modulates the microstructure and alters the dislocation distribution characteristics within the matrix, and furthermore, offers a feasible pathway for indirectly quantifying the differences in DT density across various pre-strain levels through the assessment of GND density. Regions with high GND density generally correspond to areas with high strain gradients and dislocation pile-ups, where substantial plastic deformation energy is stored. These regions are also more susceptible to becoming trap sites for hydrogen atom aggregation, thereby serving as preferential sites for crack nucleation and propagation [45,46]. The high dislocation density in the crack initiation zone is attributed to the interaction between hydrogen atoms and dislocations, which promotes dislocation multiplication and motion, while the stress concentration at the crack tip further exacerbates dislocation pile-up. The elevated dislocation density near the DTBs indicates that DTBs can act as obstacles to dislocation slip. Since the P0 specimen was not subjected to pre-strain, the density of DTs is low, and the microstructure lacks features capable of dispersing stress. As a result, cracks propagate rapidly along GBs, ultimately leading to a significant reduction in ductility.

**Figure 6:**
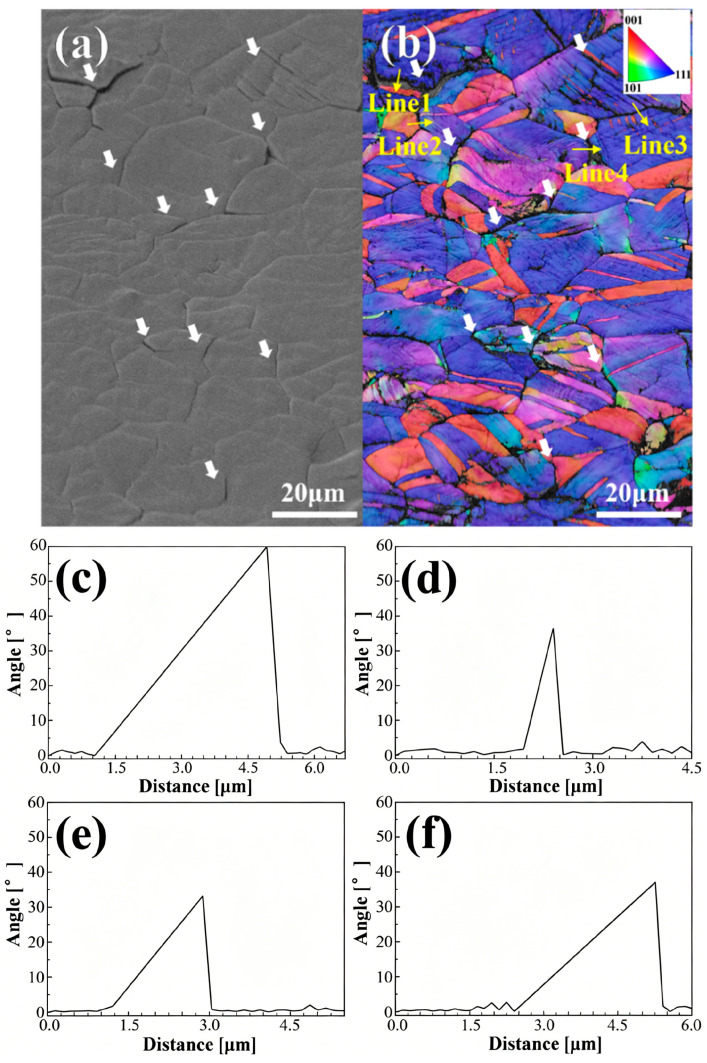
EBSD image of hydrogen induced cracks on the P50 surface: (**a**) SEM image, with white arrows showing the crack location; (**b**) IPF image, where white arrows indicate the corresponding hydrogen-induced crack positions consistent with subgraph (**a**), and the yellow lines lines 1–4 represent the misorientation angle measurement paths; (**c**–**f**) misorientation angle statistics, with statistical regions corresponding to lines 1–4 in (**b**).

Figure 6a,b present the SEM morphology and EBSD-IPF image of hydrogen-induced crack formation sites in the P50 specimen, respectively. From Figure 6a, it can be observed that the hydrogen-induced cracks in the P50 specimen exhibit short and dispersed microcrack characteristics, with most cracks initiating along GBs and TBs. Combined with the IPF orientation map in Figure 6b, it is found that the highly pre-strained microstructure contains a large number of parallel or cross-distributed DT structures. These TBs form a complex “barrier network”, causing hydrogen-induced cracks to frequently interact with TBs during propagation, forcing them to change direction or terminate, thereby significantly inhibiting long-range crack propagation. From Line 1 (Figure 6c), it is found that the misorientation angle increases sharply near the crack location, reaching a maximum value of approximately 60°. This characteristic corresponds to typical TB features, indicating that hydrogen-induced cracks can initiate or propagate along TBs. As shown in Figure 6d–f, a large number of cracks propagating along HAGBs are also observed in the P50 specimen, which is similar to the behavior observed in the P0 specimen.

**Figure 7:**
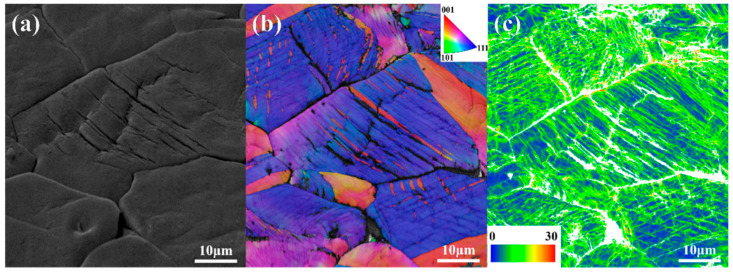
Magnified microstructural characterization of the P50 specimen after hydrogen charging and tensile testing: (**a**) SEM image; (**b**) EBSD image; and (**c**) GND density map of the P50 specimens after H-charging and tensile testing.

High-magnification SEM-EBSD characterization was performed on the P50 specimen after hydrogen charging and slow strain rate tensile testing, and the results are shown in Figure 7. Figure 7a presents a secondary electron morphology image, clearly revealing a set of parallelly distributed microcracks, whose lengths are significantly shorter than the continuous main crack observed in the P0 specimen. Figure 7b shows the EBSD-IPF image, indicating that these cracks are strictly distributed along the DTBs, consistent with the orientation of the DTBs, suggesting that hydrogen-induced cracks preferentially nucleate at DTBs and propagate along them. As typical coherent interfaces in FCC alloys, TBs serve both as effective hydrogen traps and strong obstacles to dislocation motion. Hydrogen atoms are trapped and enriched at TBs, reducing the interfacial cohesion. Meanwhile, dislocation slip is impeded at TBs, resulting in substantial dislocation pile-up at the boundary front and inducing significant local stress concentration [47,48,49]. Under the synergistic effect of hydrogen-induced interface weakening and stress concentration, microcracks preferentially initiate at TBs and propagate along the interfaces, ultimately forming the parallelly distributed crack morphology.

Figure 7c shows the GND density distribution map of the corresponding region. Similar to the P0 specimen, the GND density around the crack tips and along the crack propagation paths is significantly increased, indicating severe plastic deformation and stress concentration in these areas. However, it is worth noting that the P50 specimen also exhibits a generally higher GND density within the grain interiors, and the dislocation distribution shows characteristics of being aligned along the DTBs. This observation suggests that the high density of DTBs not only serves as crack propagation paths but also profoundly regulates the multiplication and distribution of dislocations. The presence of DTBs promotes the uniform multiplication and slip of dislocations, forming a dislocation network distributed throughout the matrix [50]. This uniformly distributed high-density dislocation network, acting in synergy with the DTBs, effectively relieves the local stress concentration at crack tips and inhibits the propagation of microcracks into long-range main cracks, thereby significantly enhancing the HE resistance of the P50 specimen.

### 3.3. Analysis of Hydrogen Distribution and Microscale Mechanical Behavior

**Figure 8:**
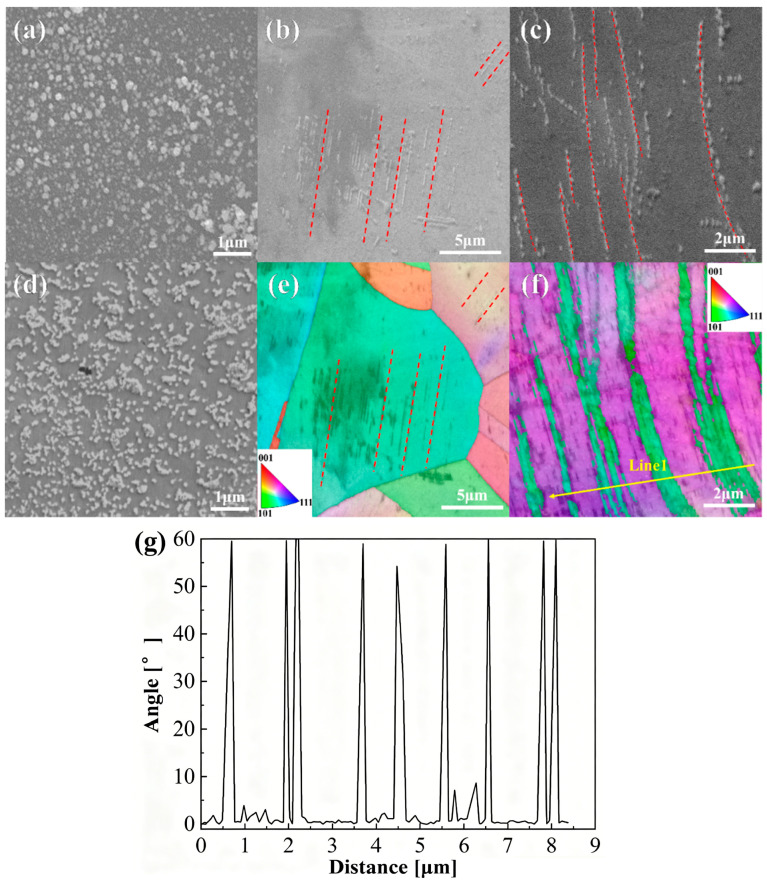
SEM images of Ag particles on (**a**) the P0 and (**d**) P50 specimens without tensile test; (**b**) SEM image and (**e**) EBSD-IPF image of the Ag particles on P0 after 10% engineering strain; (**c**) SEM image and (**f**) EBSD-IPF image of the Ag particles on the P50 specimen after 10% engineering strain. The red dashed lines in (**b**,**c**,**e**,**f**) denote plastic strain localization bands; line 1 in (**f**) is the test path for misorientation angle measurement; (**g**) measurement results of misorientation for line 1 in (**f**).

Figure 8a,d display the HMP results of the P0 and P50 specimens without applied strain, respectively. It can be observed that silver particles are uniformly and dispersedly distributed on the surface of both specimens without obvious local aggregation. Such homogeneous hydrogen distribution is attributed to the inherent lattice distortion effect of MPEAs. These distortion sites act as effective hydrogen trapping sites to capture interstitial hydrogen atoms, restrain the long-range diffusion and local segregation of hydrogen, and thereby enable hydrogen atoms to distribute evenly inside grains and prevent early hydrogen accumulation [39,40]. After the P0 specimen was subjected to 10% engineering strain, the distribution pattern of silver particles transformed from uniform dispersion into parallel band-shaped aggregation, as shown in Figure 8b. Combined with the EBSD-IPF results in Figure 8e, these silver-enriched parallel bands are located inside grains and highly consistent with the orientation of slip bands. The HMP technique visualizes hydrogen distribution via a redox reaction between silver ions and hydrogen atoms escaping the specimen surface, which generates precipitated silver particles. Consequently, silver particle patterns only reveal the steady-state spatial accumulation of hydrogen instead of real-time dynamic hydrogen transport within the matrix [51,52]. Minor hydrogen loss and quantitative discrepancies may occur during specimen handling. However, uniform experimental parameters for all specimens guarantee credible relative hydrogen distribution trends. The banded silver enrichment phenomenon directly verifies that hydrogen atoms migrate along with dislocations which serve as critical hydrogen carriers during plastic deformation. Hydrogen atoms are released when dislocations move toward the specimen surface, and subsequently react with silver ions to form silver precipitates [53]. This finding is in good agreement with the hydrogen-induced cracking behavior of the P0 specimen. During deformation, hydrogen atoms migrate with moving dislocations and gradually accumulate at GBs, which induces severe local stress concentration and further promotes the initiation and propagation of intergranular hydrogen-induced cracks, as illustrated in Figure 4 and Figure 5.

After the P50 specimen was subjected to 10% engineering strain, silver particles also presented a parallel band-like distribution, as shown in Figure 8c. Nevertheless, combined with EBSD-IPF characterization in Figure 8f and misorientation statistical results in Figure 8g, substantial differences in essential hydrogen enrichment behavior can be identified between P50 and P0 specimens. Silver particles preferentially accumulate at interfaces with distinct crystallographic orientations in the P50 specimen (Figure 8f). The line-scan misorientation results reveal that these parallel silver-enriched bands correspond to sharp misorientation peaks of approximately 60° (Figure 8g), which is a typical crystallographic feature of [111]-type DTBs in FCC alloys. This confirms that hydrogen atoms are predominantly enriched along DTBs in the strained P50 specimen. Unlike hydrogen migration toward GBs via dislocation motion in the P0 specimen, abundant dense DTBs in the P50 specimen act as strong hydrogen traps to preferentially capture and immobilize hydrogen atoms, thereby inhibiting hydrogen migration toward vulnerable GBs. Such interfacial hydrogen locking effect fundamentally restrains hydrogen aggregation and resultant stress concentration at GBs, which serves as one of the core mechanisms responsible for the prominently enhanced HE resistance of the P50 alloy. It is noteworthy that partial diffusible hydrogen may escape from the specimen surface during plastic deformation, leading to a slightly lower deposition density of silver particles on strained specimens compared with undeformed counterparts. Nevertheless, this phenomenon exerts negligible influence on the qualitative analysis of hydrogen distribution characteristics [53].

**Figure 9:**
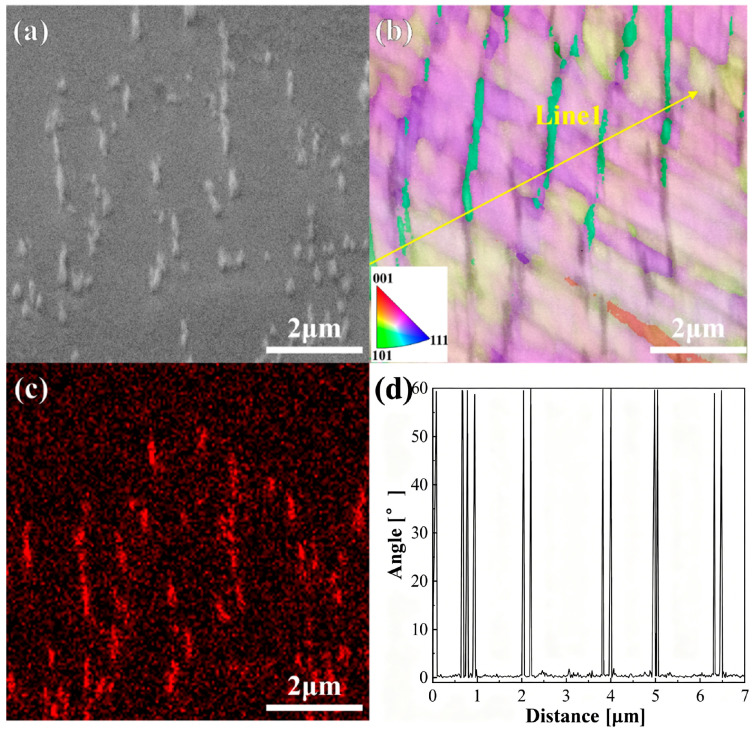
EBSD and EDS analyses of the deformed P50 specimen: (**a**) SEM image of the HMP results of P50 after H-charging with 10% engineering strain in another region; (**b**) EBSD-IPF diagram, where line 1 denotes the linear path for misorientation angle measurement; (**c**) EDS diagram; (**d**) measured misorientation of line 1 in (**b**).

Figure 9 presents the HMP results of the hydrogen-charged P50 specimen in another region. In Figure 9a,b, the discontinuous line-segment distribution of Ag particles along DTBs again confirms that hydrogen atoms are primarily enriched at DTBs in the P50 specimen during plastic deformation, which is consistent with the results shown in Figure 8c. The energy dispersive spectroscopy (EDS) mapping in Figure 9c verifies that the line-shaped particles are metallic Ag, which aligns with the fundamental principle of HMP and further confirms the segregation of hydrogen atoms at TBs [51,52]. The line-scan misorientation profile in Figure 9d exhibits sharp peaks at approximately 60°, confirming that the Ag-enriched interfaces are typical [111]-type DTBs in FCC alloys. Notably, the DTs shown in Figure 9 are significantly thinner, with the thinnest lamellae measuring only a few tens of nanometers.

**Figure 10:**
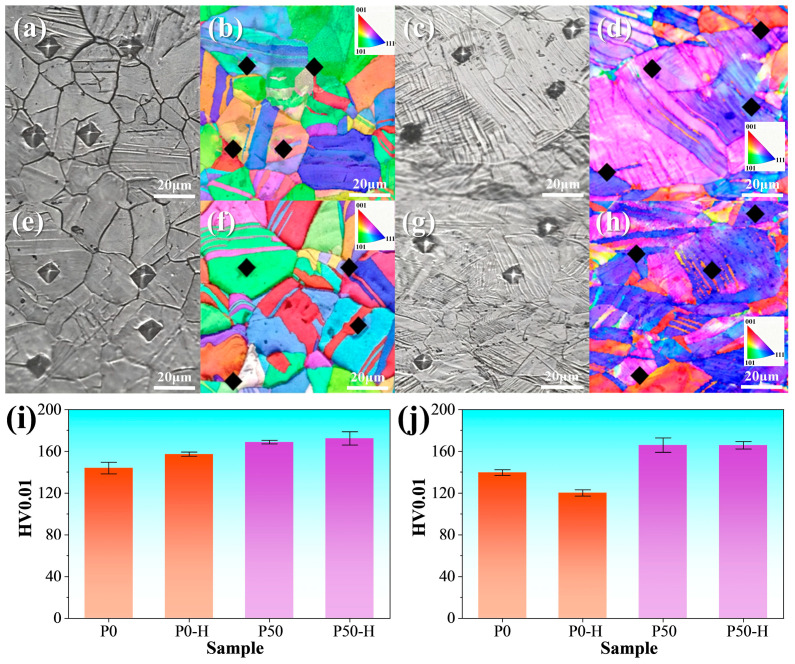
Microhardness characterization of grain interiors and GBs in P0 and P50 specimens under 10% engineering strain, with and without hydrogen charging: (**a**) OM and (**b**) EBSD images of the hardness tests of the P0 specimen; (**c**) OM and (**d**) EBSD images of the hardness tests of the P50 specimen; (**e**) OM and (**f**) EBSD images of the hardness tests of the P0 specimen after H-charging; (**g**) OM and (**h**) EBSD images of the hardness tests of the P50 specimen after H-charging; (**i**) statistical analysis of the HV0.01 values within different specimen grains; (**j**) statistical analysis of the HV0.01 values at the GBs of different specimens. Black solid squares mark the positions of Vickers microhardness indentations.

Figure 10 presents the in situ microhardness test results of two typical microstructural regions (grain interiors and GBs) in the P0 and P50 specimens before and after hydrogen charging, both subjected to 10% engineering strain. The black diamond-shaped markers in panels Figure 10a–h indicate the hardness indentation locations. Specifically, Figure 10a,b,e,f show the optical microscopy (OM) images and corresponding EBSD-IPF maps of the P0 specimen before and after hydrogen charging, respectively. Figure 10c,d,g,h show the corresponding characterization results for the P50 specimen before and after hydrogen charging, respectively. The statistical results of the microhardness within grain interiors (Figure 10i) indicate that the hardness of the P0 specimen increases slightly after hydrogen charging. This can be attributed to the hydrogen-induced twinning effect. Under applied stress, solute hydrogen reduces the critical stress for twin nucleation, promoting the formation of additional DTs within the grains and thereby yielding a strengthening effect [14,54,55]. In contrast, the grain interior hardness of the P50 specimen is significantly higher than that of the P0 specimen both before and after hydrogen charging, with only a minor increase observed after charging. This is because the high pre-strain has already introduced a high density of DTs and dislocation substructures, resulting in pronounced work hardening and TB strengthening that overshadow any additional hydrogen-induced strengthening effect.

The statistical results of GB hardness (Figure 10j) reveal distinct hydrogen-induced interfacial behaviors between the two specimens. For the P0 specimen, the GB hardness decreases significantly after hydrogen charging, indicating severe interfacial weakening caused by hydrogen enrichment at GBs. This is directly related to the transport of hydrogen atoms to GBs by moving dislocations, which weakens the interfacial bonding strength. In contrast, the GB hardness of the P50 specimen shows no obvious change before and after hydrogen charging, suggesting no hydrogen-induced GB weakening. This is primarily attributed to the strong hydrogen-trapping effect of the high-density DTBs, which inhibits hydrogen migration and segregation at GBs, thereby maintaining the mechanical stability of the GBs.

## 4. Discussion

**Figure 11:**
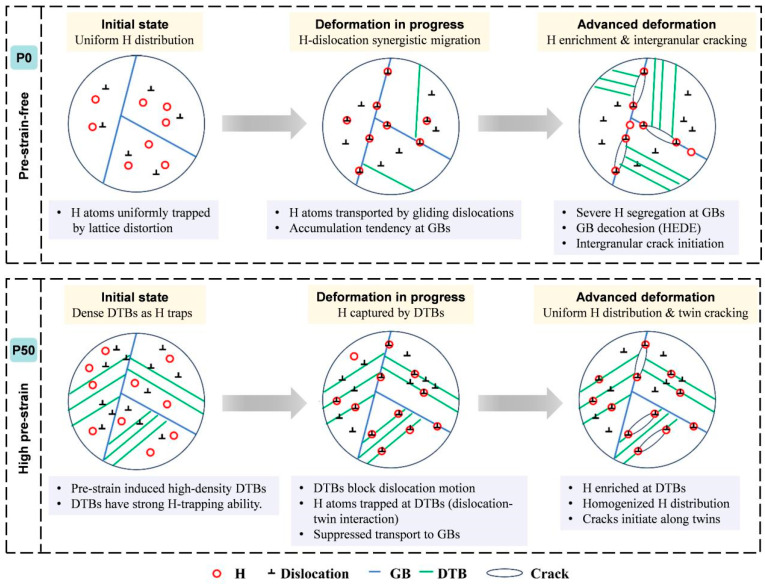
Schematic diagram showing hydrogen transport, trapping, and cracking mechanisms during deformation of the P0 (**top**) and P50 (**bottom**) specimens after H-charging.

In recent years, the regulatory mechanism of DTs on HE behavior of high/medium entropy alloys has remained controversial, and the interaction between DTBs and hydrogen atoms is one of the core factors determining the macroscopic HE resistance of materials. Combined with the schematic mechanism diagram in Figure 11 and previous experimental results, the distinct hydrogen migration pathways and hydrogen-induced damage behaviors of P0 and P50 specimens were systematically clarified, and the inhibition mechanism of pre-strain-induced DTs on HE was further elaborated. As illustrated in the upper part of Figure 11, the migration behavior of hydrogen atoms in the pre-strain-free P0 specimen is completely governed by dislocation motion during plastic deformation. Initially, hydrogen atoms are uniformly dispersed owing to the trapping effect of intrinsic lattice distortion, which corresponds to the homogeneous distribution of Ag particles in undeformed specimens revealed by HMP results (Figure 8a). During subsequent deformation, hydrogen atoms migrate towards GBs along with gliding dislocations and eventually accumulate severely at GBs. The TDS statistical results are shown in Table S7. The P0 specimen exhibits the lowest total hydrogen content, measured at 7.49 ± 0.82 ppm. Nevertheless, the specimen lacks strong hydrogen traps such as high-density dislocations and TBs, enabling trace hydrogen to diffuse along dislocation channels and heavily segregate at GBs, which weakens interfacial bonding and triggers intergranular cracking. This evidence demonstrates that the location and existing state of hydrogen dominate HE damage far more than total hydrogen concentration. This process directly activates the HEDE mechanism, in which hydrogen segregation weakens interatomic bonding at GBs and induces irreversible deterioration in interfacial mechanical properties, which is consistent with the remarkable reduction in GB hardness of hydrogen-charged P0 specimens (Figure 10j). The sequential reaction of hydrogen-dislocation synergistic migration, hydrogen enrichment at GBs and interfacial softening serves as the fundamental origin for the initiation and propagation of intergranular hydrogen-induced cracks, which is highly consistent with the intergranular fracture characteristics of P0 specimens observed via SEM and EBSD characterization (Figure 4 and Figure 5). Although hydrogen charging can induce the formation of a small number of DTs inside grains and exert a certain matrix strengthening effect via hydrogen-induced twinning (Figure 10i), such strengthening effect is far insufficient to offset the adverse influence caused by GB weakening, thus failing to fundamentally restrain the occurrence of hydrogen-induced cracking.

In contrast, high-density DTBs introduced by high pre-strain fundamentally alter the migration mode and distribution characteristic of hydrogen atoms in P50 specimens. As displayed in the lower part of Figure 11, HMP results confirm that the main hydrogen enrichment sites shift from GBs to DTBs in P50 specimens (Figure 8c,f, and Figure 9a,b), forming a unique priority hydrogen trapping behavior at DTBs. First, this results from the high stress concentration generated at the tip of a moving DT when it encounters an obstructing TB. This stress concentration creates a local strain field that attracts and immobilizes hydrogen atoms at the TB. This also partly explains why hydrogen-induced microcracks preferentially initiate along DTBs in the P50 specimens (Figure 7). In addition, this arises from the dislocation blocking effect of TBs and their ability to immobilize hydrogen. Acting as powerful obstacles to dislocation motion, TBs can effectively hinder the glide of mobile dislocations. When perfect dislocations arrive at TBs, they dissociate at the interface as following reaction [56]: $1/2[\bar{1}01](111)\to 1/6[\bar{1}14](\bar{5}\bar{1}\bar{1})+1/6[\bar{2}\bar{1}\bar{1}](\bar{1}11)$. One resultant partial dislocation can penetrate the TB and propagate into the twin interior, while the other remains pinned at the boundary as an interfacial partial dislocation [57]. This residual partial dislocation introduces slip steps along TBs [58], disrupting the long-range ordering of the coherent TBs and inducing local lattice distortion and strain fields. During the plastic deformation stage, high-density nanotwin boundaries persistently impede gliding dislocations, causing substantial dislocation pile-up and the multiplication of GNDs ahead of the interfaces. The low SFE facilitates full dissociation of perfect dislocations, thereby distributing plastic deformation across multiple twin lamellae and effectively suppressing localized stress concentrations. Meanwhile, TBs serve as long-term storage sites for various dislocation types, significantly enhancing the work-hardening capacity, while hydrogen further contributes to this hardening by promoting the extensive generation of partial dislocations through enhanced dislocation–TB interactions [59]. In contrast, specimens subjected to low pre-strain (P30) contain only a sparse population of twins [31], which precludes the formation of a globally uniform dislocation–twin interaction network. The distorted interfaces induced by such dislocation-twin interactions serve as effective hydrogen traps, enabling more diffusible hydrogen to spontaneously accumulate and become captured at the TBs [60,61]. This mechanism is directly corroborated by the GND density distribution maps of the P50 specimen. Unlike the localized increases in GND density observed near crack tips and TBs in the P0 specimen (Figure 5c,d), the P50 specimen exhibits a generally elevated GND density throughout the grain interior, with dislocations arranged along DTBs to form a dislocation network that pervades the matrix (Figure 7c). This indicates that DTBs not only serve as pathways for crack propagation but also profoundly regulate dislocation multiplication and redistribution, promoting the formation of a homogeneous dislocation network that effectively alleviates local stress concentrations at crack tips. Additionally, hydrogen segregated at TBs can promote the dissociation of perfect dislocations ahead of TBs by lowering the SFE [21,22] and the nucleation energy of partial dislocations [62,63], thereby forming more stable partial dislocation configurations. Simultaneously, this hydrogen segregation enhances the resistance of TBs to dislocation slip transfer (the passivation effect) [64], enabling plastic deformation to be distributed more uniformly across multiple slip systems and consequently improving overall plasticity homogeneity. The CoCrNi MEA possesses a low SFE of approximately 22 mJ m^−2^, which facilitates the formation of nano-sized DTs with thickness ranging from tens to hundreds of nanometers during plastic deformation [64,65,66]. Such DTs contain a large number of dislocations and nanotwin boundaries, resulting in a larger specific surface area and more severe lattice distortion, thereby exhibiting a stronger hydrogen trapping capability [15]. This effectively prevents the long-range migration of hydrogen atoms to GBs, mitigates the heterogeneity of hydrogen distribution, and eliminates both hydrogen-induced segregation at GBs and the associated stress concentrations. Therefore, based on the TDS results, although the P50 specimen exhibits the highest total hydrogen content (10.09 ± 1.04 ppm) (Table S7), the high-density TBs can serve as potent hydrogen traps and diffusion barriers, confining hydrogen in the vicinity of the TBs and effectively blocking its long-range transport to GBs, thereby preventing significant degradation of the mechanical properties at the GBs. This conclusion is fully supported by the test results showing no appreciable change in the GB hardness of the P50 specimen before and after hydrogen charging (Figure 10j).

It is noteworthy that the plastic deformation of CoCrNi MEA is dominated by the synergistic effect of dislocation slip and mechanical twinning. A large number of slip systems and dislocations are generated at the early deformation stage of P0 specimens, providing rapid diffusion channels for hydrogen transportation towards GBs. Combined with the TDS-measured results showing an increase in hydrogen content with increasing pre-strain, it is indicated that pre-straining introduces a large number of dislocations and TBs that can serve as hydrogen traps. While these traps raise the total amount of hydrogen captured, more importantly, they fundamentally alter the distribution pattern of hydrogen. The P30 specimen contains a high density of dislocations but only a limited number of DTs, and its HE susceptibility, as reflected by a ductility loss of 11.51%, is comparable to that of P0 (12.67%) [31]. This indicates that dislocation accumulation alone is insufficient to effectively impede the migration of hydrogen toward GBs. In contrast, the pre-existing dense DTBs in P50 preferentially trap diffusible hydrogen, thereby avoiding the dual stress concentration risks associated with both TBs and GBs during subsequent deformation. The hydrogen trapping effect dominated by dense DTBs fundamentally blocks the pathway of hydrogen-induced interfacial deterioration and prominently improves the HE resistance of the alloy. This strategy realizes the double regulation of homogenized hydrogen distribution and reduced stress concentration sites, and consequently effectively inhibits the initiation and propagation of hydrogen-induced cracks.

It should be noted that this study has preliminarily revealed how pre-strain-induced twins regulate hydrogen migration and hydrogen-induced damage using TDS, HMP, EBSD, and mechanical testing. Future study will employ transmission electron microscopy (TEM), EBSD, and atom probe tomography (APT) to systematically characterize the type, density, and spatial distribution of hydrogen traps, along with TDS peak deconvolution, to establish the quantitative relationship between trap types (diffusible hydrogen, dislocations, GBs and TBs) and desorption peak temperatures. This will further elucidate the complete mechanism from hydrogen trapping and migration to damage initiation. Additionally, validation under long-term exposure to 100 MPa gaseous hydrogen and at lower strain rates (10^−6^–10^−7^ s^−1^) is required, along with in situ neutron diffraction or deuterium tracing techniques to directly track the real-time partitioning of hydrogen between TBs and GBs. Meanwhile, the main trade-off of this strategy is reflected in the reduction in macroscopic ductility. Future study may address this issue by optimizing the pre-strain level (e.g., 40%) or introducing post-deformation annealing treatments to finely tune the twin density and dislocation configuration, thereby further restoring ductility while maintaining high strength and excellent hydrogen embrittlement resistance, and achieving a more favorable balance among the three properties.

## 5. Conclusions

Pre-straining effectively tailors the microstructural evolution of CoCrNi MEAs and significantly enhances their resistance to HE. The 50% pre-strained specimen (P50), featuring a high density of nanoscale DTs, exhibited only 2.74% elongation loss after hydrogen charging, far outperforming the unstrained (P0) and 30% pre-strained (P30) specimens. In P0, during plastic deformation, hydrogen migrates along dislocations toward GBs, activating HEDE, which irreversibly degrades GB mechanical properties and triggers typical intergranular hydrogen-induced cracking. This mechanism was corroborated by EBSD and microhardness measurements. In contrast, the dense nanoscale TBs in P50 fundamentally alter hydrogen transport and distribution, acting as efficient traps that capture and immobilize hydrogen, thereby suppressing long-range diffusion and segregation to GBs and eliminating the risk of GB weakening at the source. Simultaneously, DTBs strongly impede dislocation motion, promoting the dissociation of perfect dislocations into Shockley partials and forming dense dislocation networks at twin interfaces. Combined with the low SFE of CoCrNi MEA, this promotes uniform hydrogen distribution, mitigates local stress concentrations, and effectively suppresses crack initiation and propagation. This study systematically elucidates the microstructural mechanisms by which pre-strain-induced nanoscale twins enhance HE resistance in CoCrNi MEAs, providing theoretical guidance and practical insights for microstructural design of high-performance MPEAs for hydrogen energy storage, transport, and low-temperature engineering applications.

Based on the hydrogen-twin interaction mechanism proposed in this study, future research will be carried out in three main directions: (1) measuring twin-boundary hydrogen trap binding energy using variable-rate TDS and APT; (2) observing dislocation dissociation at TBs via in situ TEM to clarify dislocation-twin mechanisms; and (3) conducting micro-pillar compression tests in hydrogen environments to quantify twin-boundary cracking stress and validate the hydrogen passivation mechanism. Pre-straining to introduce nano-twins is a practical strategy for improving HE resistance. In hydrogen energy systems, liquid hydrogen tanks and pipelines operate under hydrogen-rich conditions and require high resistance to hydrogen damage. The approach demonstrated here, which uses pre-straining to generate high-density nano-twins, provides a processing reference for CoCrNi MEAs in cryogenic hydrogen storage, and is also broadly applicable to other low-SFE FCC alloys such as austenitic stainless steels and high-Mn TWIP steels, making it relevant to a wide range of structural material systems.

## Data Availability

The original contributions presented in this study are included in the article/Supplementary Materials. Further inquiries can be directed to the corresponding author.

## Supplementary Materials

The following supporting information can be downloaded at https://www.mdpi.com/article/10.3390/ma19143010/s1: Figure S1. Engineering stress–strain curves of P0, P30, and P50 and their hydrogen-charged counterparts, showing the results of three independent repeated tests, respectively: (a) P0; (b) P30; (c) P50; (d) P0-H; (e) P30-H; (f) P50-H. Table S1. Statistical results of key mechanical performance parameters for the P0 specimens. Table S2. Statistical results of key mechanical performance parameters for the P30 specimens. Table S3. Statistical results of key mechanical performance parameters for the P50 specimens. Table S4. Statistical results of key mechanical performance parameters for the P0-H specimens. Table S5. Statistical results of key mechanical performance parameters for the P30-H specimens. Table S6. Statistical results of key mechanical performance parameters for the P50-H specimens. Table S7. Statistical results of hydrogen content variation with pre-strain level after 48 h of hydrogen charging. References [67,68,69] are cited in the Supplementary Materials.

## Nomenclature

Acronyms	
HE	Hydrogen embrittlement
MEA	Medium-entropy Alloy
MPEA	Multi-principal element alloy
FCC	Face-centered cubic
SFE	Stacking fault energy
GB	Grain boundary
TB	Twin boundary
DT	Deformation twin
DTB	Deformation twin boundary
HAGB	High-angle grain boundary
LAGB	Low-angle grain boundary
HEDE	Hydrogen-enhanced decohesion mechanism
HELP	Hydrogen-enhanced localized plasticity
GND	Geometrically necessary dislocation
SSRT	Slow strain rate tensile (test)
EBSD	Electron backscatter diffraction
HMP	Hydrogen microprinting
TDS	Thermal desorption spectroscopy
SEM	Scanning electron microscope/microscopy
IPF	Inverse pole figure
EDS	Energy dispersive spectroscopy
OM	Optical microscopy
EBSD-IPF	EBSD Inverse Pole Figure
Specimen Designations	
P0	0% pre-strained specimen
P30	30% pre-strained specimen
P50	50% pre-strained specimen
H-charged	Hydrogen-charged specimen
H-free	Hydrogen-free (uncharged) specimen
Parameters and Variables	
*EI_loss_*	Total elongation loss (HE susceptibility index)
*EI_free_*	Tensile elongation of H-free specimen
*EI_H_*	Tensile elongation of H-charged specimen
*HV*_0.01_	Vickers hardness at 0.01 kg load
*σ*	Engineering stress MPa
*ε*	Engineering strain %
*θ*	Grain boundary misorientation angle
Crystallographic Notations	
[111]	Crystallographic plane index (twin plane in FCC alloys)
$1/2[\bar{1}01]$	Perfect dislocation Burgers vector and slip plane
$1/6[\bar{1}14](\bar{5}\bar{1}\bar{1})$	Shockley partial dislocation (type 1)
$1/6[\bar{2}\bar{1}\bar{1}](\bar{1}11)$	Shockley partial dislocation (type 2)

## References

[1] Dwivedi S.K., Vishwakarma M. (2018). Hydrogen embrittlement in different materials: A review. Int. J. Hydrogen Energy.

[2] Gludovatz B., Hohenwarter A., Thurston K.V., Bei H., Wu Z., George E.P., Ritchie R.O. (2016). Exceptional damage-tolerance of a medium-entropy alloy CrCoNi at cryogenic temperatures. Nat. Commun..

[3] Zhang D., Zhang J., Kuang J., Liu G., Sun J. (2021). Superior strength-ductility synergy and strain hardenability of Al/Ta co-doped NiCoCr twinned medium entropy alloy for cryogenic applications. Acta Mater..

[4] Li S., Wu F., Zhang Y., Zhou R., Lu Z., Jiang Y., Bian T., Shang D., Zhang L. (2024). Enhanced hydrogen storage performance of magnesium hydride catalyzed by medium-entropy alloy CrCoNi nanosheets. Int. J. Hydrogen Energy.

[5] Liu D., Yu Q., Kabra S., Jiang M., Forna-Kreutzer P., Zhang R., Payne M., Walsh F., Gludovatz B., Asta M. (2022). Exceptional fracture toughness of CrCoNi-based medium-and high-entropy alloys at 20 kelvin. Science.

[6] Ueda T., Yoshida S., Dangwal S., Gondek Ł., Fukami K., Kučerová L., Saksl K., Edalati K., Tsuji N. (2025). Effect of lattice defects on hydrogen storage properties of HfNbTiZr medium entropy alloy. Int. J. Hydrogen Energy.

[7] Xu D., Wang M., Li T., Wei X., Lu Y. (2022). A critical review of the mechanical properties of CoCrNi-based medium-entropy alloys. Microstructures.

[8] Wang R., Hu L., Geng P., Zhang W., Du C. (2025). Deformation and strengthening mechanism of non-equiatomic CoCrNi medium entropy alloys. Mater. Sci. Eng. A.

[9] Li Q., Mo J., Ma S., Duan F., Zhao Y., Liu S., Liu W., Zhao S., Liu C., Liaw P. (2022). Defeating hydrogen-induced grain-boundary embrittlement via triggering unusual interfacial segregation in FeCrCoNi-type high-entropy alloys. Acta Mater..

[10] Fu Z., Yang B., Chen M., Gou G., Chen H. (2021). Effect of recrystallization annealing treatment on the hydrogen embrittlement behavior of equimolar CoCrFeMnNi high entropy alloy. Int. J. Hydrogen Energy.

[11] Chung D.H., Kim Y.K., Kim Y.K., Song S.Y., Kwon H.J., Sohn S.S., Na Y.S. (2023). Enhancing hydrogen embrittlement resistance of medium entropy alloys by forming dislocation cell walls. Corros. Sci..

[12] Ye F., Chen W., Wu H., Zhu T., Zhang P., Xu Q., Cao X. (2025). Effect of L12 precipitates on hydrogen-induced defects and hydrogen embrittlement behavior in CoCrFeNi-based high-entropy alloys. J. Alloys Compd..

[13] Marques S.C., dos Santos D.S. (2024). The role of precipitation in hydrogen diffusivity and mechanical properties of a high entropy alloy. Int. J. Hydrogen Energy.

[14] Luo H., Lu W., Fang X., Ponge D., Li Z., Raabe D. (2018). Beating hydrogen with its own weapon: Nano-twin gradients enhance embrittlement resistance of a high-entropy alloy. Mater. Today.

[15] Koyama M., Akiyama E., Tsuzaki K., Raabe D. (2013). Hydrogen-assisted failure in a twinning-induced plasticity steel studied under in situ hydrogen charging by electron channeling contrast imaging. Acta Mater..

[16] So K.H., Kim J.S., Chun Y.S., Park K.-T., Lee Y.-K., Lee C.S. (2009). Hydrogen delayed fracture properties and internal hydrogen behavior of a Fe–18Mn–1.5 Al–0.6 C TWIP steel. ISIJ Int..

[17] Ryu J.H., Kim S.K., Lee C.S., Suh D.-W., Bhadeshia H. (2013). Effect of aluminium on hydrogen-induced fracture behaviour in austenitic Fe–Mn–C steel. Proc. R. Soc. A Math. Phys. Eng. Sci..

[18] Laplanche G., Kostka A., Horst O., Eggeler G., George E. (2016). Microstructure evolution and critical stress for twinning in the CrMnFeCoNi high-entropy alloy. Acta Mater..

[19] Bouaziz O., Allain S., Scott C. (2008). Effect of grain and twin boundaries on the hardening mechanisms of twinning-induced plasticity steels. Scr. Mater..

[20] Yi J., Zhuang X., He J., He M., Liu W., Wang S. (2021). Effect of Mo doping on the gaseous hydrogen embrittlement of a CoCrNi medium-entropy alloy. Corros. Sci..

[21] Zhou X.-Y., Wu H.-H., Zhou M., Wang L., Lookman T., Mao X. (2025). Enhanced hydrogen embrittlement resistance of FeCoNiCrMn multi-principal element alloys via local chemical ordering and grain boundary segregation. Acta Mater..

[22] Cheng H., Luo H., Pan Z., Wang X., Zhao Q., Fu Y., Li X. (2023). Effects of laser powder bed fusion process parameters on microstructure and hydrogen embrittlement of high-entropy alloy. J. Mater. Sci. Technol..

[23] Zhu Q., Wei S., Zhang Q., Zhao Y., Ramamurty U., Lu Y., Gao H. (2025). Hydrogen-induced twin boundary passivation in multi-principal element alloy: A micropillar compression study. Int. J. Plast..

[24] Liu S., Xu Z., Zhu Y., Shi R., Gao K., Pang X. (2024). Superior hydrogen embrittlement resistance of CoCrNi-based medium-entropy alloy via coherent precipitation and grain boundary strengthening. Corros. Sci..

[25] Yan S., He X., Zhu Z. (2023). Hydrogen embrittlement of CrCoNi medium-entropy alloy with millimeter-scale grain size: An in situ hydrogen charging study. Entropy.

[26] Ding C., Jiao Z., Luan J., Xu B., Li R., Cao B., Zhou C., Liu W. (2024). Suppressing hydrogen embrittlement of a CrCoNi medium-entropy alloy by triggering co-segregation of carbon, boron, and Cr. Corros. Sci..

[27] Soundararajan C.K., Luo H., Raabe D., Li Z. (2020). Hydrogen resistance of a 1 GPa strong equiatomic CoCrNi medium entropy alloy. Corros. Sci..

[28] Lu N., Zhang C.-H. (2026). Mechanism of hydrogen-induced vacancy cluster decomposition in CrCoNi medium-entropy alloy. Int. J. Hydrogen Energy.

[29] Rhode M., Wetzel A., Ozcan O., Nietzke J., Richter T., Schröpfer D. (2020). Hydrogen Diffusion and Local Volta Potential in High-and Medium-Entropy Alloys. IOP Conf. Ser. Mater. Sci. Eng..

[30] An Y.H., Jung J.Y., Jung H., Kim Y.S., Lee S.Y., Lee D.-H. (2024). Effect of stress level on hydrogen-induced nanohardness variations in CoCrNi medium-entropy alloy. Mater. Charact..

[31] Wang Z., Jing S., Yan Y. (2025). Effect of Pre-Strain Induced Microstructure Evolution on Hydrogen Embrittlement Resistance of a CoCrNi Medium-Entropy Alloy. Materials.

[32] Chisholm C., Bei H., Lowry M., Oh J., Asif S.S., Warren O., Shan Z., George E.P., Minor A.M. (2012). Dislocation starvation and exhaustion hardening in Mo alloy nanofibers. Acta Mater..

[33] Arasaratnam P., Sivakumaran K., Tait M. (2011). True stress-true strain models for structural steel elements. Int. Sch. Res. Not..

[34] Wang H., Koyama M., Hojo T., Akiyama E. (2021). Hydrogen embrittlement and associated surface crack growth in fine-grained equiatomic CoCrFeMnNi high-entropy alloys with different annealing temperatures evaluated by tensile testing under in situ hydrogen charging. Int. J. Hydrogen Energy.

[35] Kwon Y.J., Lee T., Lee J., Chun Y.S., Lee C.S. (2015). Role of Cu on hydrogen embrittlement behavior in Fe–Mn–C–Cu TWIP steel. Int. J. Hydrogen Energy.

[36] Gong P., Nutter J., Rivera-Diaz-Del-Castillo P., Rainforth W. (2020). Hydrogen embrittlement through the formation of low-energy dislocation nanostructures in nanoprecipitation-strengthened steels. Sci. Adv..

[37] Ding Y., Yu H., Zhao K., Lin M., Xiao S., Ortiz M., He J., Zhang Z. (2021). Hydrogen-induced transgranular to intergranular fracture transition in bi-crystalline nickel. Scr. Mater..

[38] Takasawa K., Ikeda R., Ishikawa N., Ishigaki R. (2012). Effects of grain size and dislocation density on the susceptibility to high-pressure hydrogen environment embrittlement of high-strength low-alloy steels. Int. J. Hydrogen Energy.

[39] Jothi S., Merzlikin S., Croft T., Andersson J., Brown S. (2016). An investigation of micro-mechanisms in hydrogen induced cracking in nickel-based superalloy 718. J. Alloys Compd..

[40] Wang D., Lu X., Wan D., Guo X., Johnsen R. (2021). Effect of hydrogen on the embrittlement susceptibility of Fe–22Mn-0.6 C TWIP steel revealed by in-situ tensile tests. Mater. Sci. Eng. A.

[41] Ogawa Y., Umakoshi K., Nakamura M., Takakuwa O., Matsunaga H. (2020). Hydrogen-assisted, intergranular, fatigue crack-growth in ferritic iron: Influences of hydrogen-gas pressure and temperature variation. Int. J. Fatigue.

[42] Zhi H., Zhang C., Antonov S., Yu H., Guo T., Su Y. (2020). Investigations of dislocation-type evolution and strain hardening during mechanical twinning in Fe-22Mn-0.6 C twinning-induced plasticity steel. Acta Mater..

[43] Meng Z., Yang M., Feng A., Qu S., Zhao F., Yang L., Yao J., Yang Y., Fan Q., Wang H. (2023). Transfer or blockage: Unraveling the interaction between deformation twinning and grain boundary in tantalum under shock loading with molecular dynamics. J. Mater. Sci. Technol..

[44] Fu H., Wang W., Zhao H., Jin F., Li J. (2020). Study of hydrogen-induced delayed fracture in high-Mn TWIP/TRIP steels during in situ electrochemical hydrogen-charging: Role of microstructure and strain rate in crack initiation and propagation. Corros. Sci..

[45] Zhi H., Antonov S., Zhang C., Guo Z., Su Y. (2020). Origins of back stress strengthening in Fe-22Mn-0.6 C (-3Al) TWIP steels. Mater. Sci. Eng. A.

[46] Zhi H., Zhang C., Guo Z., Antonov S., Su Y. (2020). Outstanding tensile properties and their origins in twinning-induced plasticity (TWIP) steels with gradient substructures. Materials.

[47] Michler T., Naumann J. (2010). Hydrogen embrittlement of Cr-Mn-N-austenitic stainless steels. Int. J. Hydrogen Energy.

[48] Koyama M., Akiyama E., Sawaguchi T., Ogawa K., Kireeva I.V., Chumlyakov Y.I., Tsuzaki K. (2013). Hydrogen-assisted quasi-cleavage fracture in a single crystalline type 316 austenitic stainless steel. Corros. Sci..

[49] Bechtle S., Kumar M., Somerday B.P., Launey M.E., Ritchie R.O. (2009). Grain-boundary engineering markedly reduces susceptibility to intergranular hydrogen embrittlement in metallic materials. Acta Mater..

[50] Shen Y., Zhang F., Li M., Li G., Zhu D., Su H., Jiang W. (2026). The effect of twin boundary spacing, orientation and separation of grain boundary affected zones on the mechanical properties of twinned Cu-Ag alloys. Appl. Phys. A.

[51] Tarzimoghadam Z., Rohwerder M., Merzlikin S.V., Bashir A., Yedra L., Eswara S., Ponge D., Raabe D. (2016). Multi-scale and spatially resolved hydrogen mapping in a Ni–Nb model alloy reveals the role of the δ phase in hydrogen embrittlement of alloy 718. Acta Mater..

[52] Perez T., Garcia J.O. (1982). Direct observation of hydrogen evolution in the electron microscope scale. Scr. Metall..

[53] Pu S., Ooi S. (2019). Hydrogen transport by dislocation movement in austenitic steel. Mater. Sci. Eng. A.

[54] Zhang C., Zhi H., Antonov S., Chen L., Su Y. (2021). Hydrogen-enhanced densified twinning (HEDT) in a twinning-induced plasticity (TWIP) steel. Scr. Mater..

[55] Nie Y.J., Yang F., Meng L.X., Wang Y.Z., Yin L., Shi Q.X., Ma J.Y., Liang W., Zheng L.W. (2024). Hydrogen-enhanced densified deformation twins in 304L austenitic stainless steel fabricated by selective laser melting. Mater. Today Commun..

[56] Mahajan S., Chin G. (1973). Twin-slip, twin-twin and slip-twin interactions in Co-8 wt.% Fe alloy single crystals. Acta Metall..

[57] Li Z., Zhang J., Zhai Y., Zhang J., Wang X., Zhang Z., Mao S., Han X. (2022). Dynamic mechanisms of strengthening and softening of coherent twin boundary via dislocation pile-up and cross-slip. Mater. Res. Lett..

[58] Wang Y., Sui M. (2009). Atomic-scale in situ observation of lattice dislocations passing through twin boundaries. Appl. Phys. Lett..

[59] Fan Y., Cui F., Lu L., Zhang B. (2019). A nanotwinned austenite stainless steel with high hydrogen embrittlement resistance. J. Alloys Compd..

[60] Lu L., Chen X., Huang X., Lu K. (2009). Revealing the maximum strength in nanotwinned copper. Science.

[61] Zhang Z., Sheng H., Wang Z., Gludovatz B., Zhang Z., George E.P., Yu Q., Mao S.X., Ritchie R.O. (2017). Dislocation mechanisms and 3D twin architectures generate exceptional strength-ductility-toughness combination in CrCoNi medium-entropy alloy. Nat. Commun..

[62] Lumbeeck G., Idrissi H., Amin-Ahmadi B., Favache A., Delmelle R., Samaee V., Proost J., Pardoen T., Schryvers D. (2018). Effect of hydriding induced defects on the small-scale plasticity mechanisms in nanocrystalline palladium thin films. J. Appl. Phys..

[63] Amin-Ahmadi B., Connétable D., Fivel M., Tanguy D., Delmelle R., Turner S., Malet L., Godet S., Pardoen T., Proost J. (2016). Dislocation/hydrogen interaction mechanisms in hydrided nanocrystalline palladium films. Acta Mater..

[64] Dong J., Li F., Gu Z., Jiang M., Liu Y., Wang G., Wu X. (2023). Impact resistance and energy dissipation mechanism of nanocrystalline CoCrNi medium entropy alloy nanofilm under supersonic micro-ballistic impact. Int. J. Plast..

[65] Xie Y., Lu T., Zhao P., Sun B., Yao N., Chen X., Tan J., Zhang X.-C., Tu S.-T. (2023). Cryoforged nanotwinned CoCrNi medium-entropy alloy with exceptional fatigue property at cryogenic temperature. Scr. Mater..

[66] Sathiyamoorthi P., Moon J., Bae J.W., Asghari-Rad P., Kim H.S. (2019). Superior cryogenic tensile properties of ultrafine-grained CoCrNi medium-entropy alloy produced by high-pressure torsion and annealing. Scr. Mater..

[67] Ali M.L. (2021). Enhanced lattice distortion, yield strength, critical resolved shear stress, and improving mechanical properties of transition-metals doped CrCoNi medium entropy alloy. RSC Adv..

[68] Wu L., Lin N., Liu R., Yan K., Hao Y., Wang W., Shi Q., Yu Y., Liu Z., Zeng Q. (2025). Effect of laser surface texturing on sliding wear performance and wear mechanism of CoCrNi and CoCrFeMnNi alloys. J. Mater. Sci..

[69] Zhao J.-Q., Tian H., Wang Z., Wang X.-J., Qiao J.-W. (2020). FCC-to-HCP phase transformation in CoCrNi_x_ medium-entropy alloys. Acta Metall. Sin. (Engl. Lett.).

