# Supplementary material for: Investigating the Mechanisms of Hydrogen Embrittlement Resistance in Pre-Strained CoCrNi Medium-Entropy Alloy via Hydrogen Migration and Trapping Behavior
Source: Materials (Basel). 2026 Jul 13;19(14):3010. doi: 10.3390/ma19143010 (PMC13413590; doi:10.3390/ma19143010)
Supplement: Supplementary file 1 [file materials-19-03010-s001.zip › materials-4366445-supplementary.pdf]

Supplementary Material for

Investigating the Mechanisms of Hydrogen Embrittlement Resistance in Pre-Strained CoCrNi Medium-Entropy Alloy via Hydrogen Migration and Trapping Behavior

Zening Wang [1], Sirui Jing [1] and Yu Yan [1,*]

Beijing Advanced Innovation Center for Materials Genome Engineering, Corrosion and Protection Center, Institute for Advanced Materials and Technology, University of Science and Technology Beijing, Beijing 100083, China; wangzeningning@163.com (Z.W.); jing_sr@163.com (S.J.)
* Correspondence: yanyu@ustb.edu.cn

1. Supplemental Notes

Figure S1 displays tensile curves obtained from equiatomic CoCrNi medium-entropy alloy (MEA) with various pre-strain levels before and after electrochemical hydrogen charging. Three replicate specimens were tested for each experimental condition. Statistical metrics including the minimum, maximum, mean, median, range, and standard deviation of all relevant mechanical parameters were calculated and summarized in Tables S1–S6. The measured mechanical property data demonstrate excellent repeatability and reproducibility. The elastic modulus of all tested specimens averages approximately 225 GPa, consistent with experimental values documented in published literature [1-3]. As a quantitative indicator of interatomic bonding strength within the alloy matrix, elastic modulus remains insensitive to both microstructural alterations induced by pre-straining and hydrogen incorporation.

As pre-strain increases from 0% to 30% and 50%, the alloy's yield strength and ultimate tensile strength increase markedly. As tabulated in Tables S1, S2, and S3, the yield strength σ_y rises from 293.79 MPa to 882.14 MPa and 1163.88 MPa, respectively. Conversely, elongation at fracture decreases drastically from 77.62% to 43.49% and 16.07%; full datasets are provided in Tables S1–S3. Simultaneously, both the strength coefficient K and strain-hardening exponent n experience substantial reductions. Specifically, the strain-hardening exponent n drops from 0.635 to 0.303 and 0.108 (Tables S1–S3). These trends confirm that pre-straining substantially elevates alloy strength via work hardening, yet significantly deteriorates the material's strain-hardening capacity and plastic deformability.

Distinct tensile curve characteristics are illustrated in Figure S1. The unstrained P0 specimen exhibits typical continuous yielding behavior with pronounced yielding features and an extended strain-hardening regime, yielding a fracture strain of roughly 80%. The 30% pre-strained P30 specimen exhibits a dramatic rise in yield strength accompanied by weakened yielding signatures, while a prominent strain-hardening stage is still retained, corresponding to a fracture strain of approximately 45%. In contrast, the heavily pre-strained P50 specimen behaves nearly as ideal plastic flow with almost fully depleted strain-hardening capacity, and its fracture strain plummets to around 15%. Following electrochemical hydrogen charging, slight reductions in yield strength and ultimate tensile strength are observed for all pre-strained groups, alongside further ductility degradation, as recorded in Tables S4–S6.

Nevertheless, the alloy's hydrogen embrittlement susceptibility shows a strong dependence on pre-strain magnitude. In particular, the un-prestrained P0 specimen undergoes the most severe mechanical deterioration (Tables S1 and S4): its ultimate tensile strength decreases by ~8.1%, and its elongation is reduced by ~12.7%. By comparison, the heavily pre-strained P50 specimen exhibits minimal property degradation (Tables S3 and S6), with only a 0.1% loss in ultimate tensile strength and merely a 2.7% drop in elongation.

The underlying mechanism accounting for this trend is as follows: high-density dislocations introduced by pre-straining function as reversible hydrogen traps to effectively sequester hydrogen atoms. This trapping effect hinders hydrogen diffusion and accumulation at hydrogen embrittlement-susceptible sites, such as grain boundaries and crack tips, thereby mitigating the harmful damage induced by hydrogen-triggered localized plasticity loss and interfacial decohesion. In summary, CoCrNi alloys subjected to high pre-strain possess superior hydrogen embrittlement resistance, which establishes a strong inverse correlation between pre-strain level and hydrogen embrittlement susceptibility.

2. Supplemental Figure

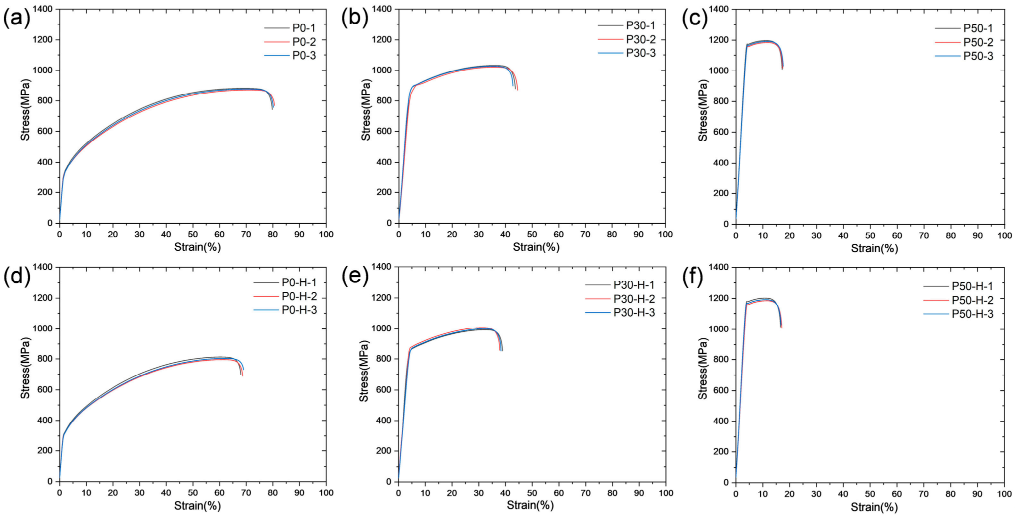

Figure S1. Engineering stress–strain curves of P0, P30, and P50 and their hydrogen-charged counterparts, showing the results of three independent repeated tests, respectively: (a) P0; (b) P30; (c) P50; (d) P0-H; (e) P30-H; (f) P50-H

3. Supplemental Table

Table S1. Statistical results of key mechanical performance parameters for the P0 specimens.

Parameter	Unit	Mean value	Standard deviation	Median value	Minimum value	Maximum value	Range
Young's modulus (*E*)	GPa	225.30	1.69	225.36	223.58	226.96	3.38
Yield strength (σ_y)	MPa	293 .79	5.44	291.94	288.25	301.18	12.93
Tensile strength (σ_b)	MPa	876.05	4.39	870.52	876.38	881.25	10.73
Strength coefficient (*K*)	MPa	2243.27	7.14	2246.21	2235.13	2248.46	13.33
Strain hardening exponent (*n*)		0.635	0.009	0.637	0.625	0.642	0.017
Fracture stress (σ_f)	MPa	757.37	8.65	758.61	748.16	765.33	17.17
Elongation (*El*)		77.62%	0.67%	77.39%	76.94%	78.53%	1.59%

Table S2. Statistical results of key mechanical performance parameters for the P30 specimens.

Parameter	Unit	Mean value	Standard deviation	Median value	Minimum value	Maximum value	Range
Young's modulus (*E*)	GPa	224.03	3.16	223.63	221.08	227.37	6.29
Yield strength (σ_y)	MPa	882.14	6.24	883.18	875.45	887.80	12.35
Tensile strength (σ_b)	MPa	1027.24	5.45	1027.40	1021.71	1032.61	10.90
Strength coefficient (*K*)	MPa	2003.70	10.90	2004.20	1992.55	2014.34	21.79
Strain hardening exponent (*n*)		0.303	0.011	0.305	0.292	0.313	0.021
Fracture stress (σ_f)	MPa	885.11	11.21	878.63	878.63	898.06	19.42
Elongation (*El*)		43.49%	1.68%	43.84%	41.66%	44.96%	3.30%

Table S3. Statistical results of key mechanical performance parameters for the P50 specimens.

Parameter	Unit	Mean value	Standard deviation	Median value	Minimum value	Maximum value	Range
Young's modulus (*E*)	GPa	224.46	2.76	223.58	222.25	227.55	5.30
Yield strength (σ_y)	MPa	1163.88	4.40	1163.99	1159.43	1168.22	8.79
Tensile strength (σ_b)	MPa	1191.10	6.26	1191.55	1184.63	1197.12	12.49
Strength coefficient (*K*)	MPa	1661.28	15.48	1665.73	1644.07	1674.05	29.98
Strain hardening exponent (*n*)		0.108	0.005	0.109	0.102	0.112	0.010
Fracture stress (σ_f)	MPa	1020.27	10.59	1018.73	1010.53	1031.55	21.02
Elongation (*El*)		16.07%	0.54%	16.01%	15.56%	16.64%	1.08%

Table S4. Statistical results of key mechanical performance parameters for the P0-H specimens.

Parameter	Unit	Mean value	Standard deviation	Median value	Minimum value	Maximum value	Range
Young's modulus (*E*)	GPa	224.18	1.92	224.39	222.16	225.98	3.82
Yield strength (σ_y)	MPa	286.59	6.39	285.93	280.56	293.28	12.72
Tensile strength (σ_b)	MPa	805.16	6.23	805.71	798.67	811.10	12.43
Strength coefficient (*K*)	MPa	2048.93	15.61	2050.83	2032.46	2063.51	31.05
Strain hardening exponent (*n*)		0.614	0.006	0.613	0.608	0.620	0.012
Fracture stress (σ_f)	MPa	704.10	15.44	700.12	691.04	721.14	30.10
Elongation (*EI*)		67.79%	0.85%	67.89%	68.58%	66.89%	1.69%

Table S5. Statistical results of key mechanical performance parameters for the P30-H specimens.

Parameter	Unit	Mean value	Standard deviation	Median value	Minimum value	Maximum value	Range
Young's modulus (*E*)	GPa	223.18	2.85	222.58	220.68	226.29	5.61
Yield strength (σ_y)	MPa	878.10	3.38	877.11	875.33	881.87	6.54
Tensile strength (σ_b)	MPa	1000.33	7.10	1000.53	993.14	1007.33	14.19
Strength coefficient (*K*)	MPa	1883.75	14.98	1878.07	1872.44	1900.74	28.30
Strain hardening exponent (*n*)		0.280	0.004	0.280	0.276	0.284	0.008
Fracture stress (σ_f)	MPa	855.09	6.85	854.30	848.66	862.30	13.64
Elongation (*EI*)		38.48%	0.93%	38.86%	37.42%	39.16%	1.74%

Table S6. Statistical results of key mechanical performance parameters for the P50-H specimens.

Parameter	Unit	Mean value	Standard deviation	Median value	Minimum value	Maximum value	Range
Young's modulus (*E*)	GPa	225.38	1.60	225.58	223.68	226.87	3.19
Yield strength (σ_y)	MPa	1161.58	5.35	1160.85	1156.63	1167.25	10.62
Tensile strength (σ_b)	MPa	1189.88	7.22	1192.58	1181.70	1195.36	13.66
Strength coefficient (*K*)	MPa	1670.14	4.21	1670.70	1665.68	1674.05	8.37
Strain hardening exponent (*n*)		0.104	0.003	0.103	0.101	0.107	0.006
Fracture stress (σ_f)	MPa	1017.25	10.37	1014.59	1008.47	1028.69	20.22
Elongation (*EI*)		15.63%	0.98%	15.52%	14.71%	16.66%	1.95%

Table S7. Statistical results of hydrogen content variation with pre-strain level after 48 h of hydrogen charging.

Parameter	Unit	Mean value	Standard deviation	Median value	Minimum value	Maximum value	Range
P0	ppm	7.49	0.82	7.89	6.55	8.03	1.48
P30	ppm	7.94	1.03	8.08	6.85	8.90	2.05
P50	ppm	10.09	1.04	10.45	8.92	10.90	1.98

References

1. Ali, M.L.; Enhanced lattice distortion, yield strength, critical resolved shear stress, and improving mechanical properties of transition-metals doped CrCoNi medium entropy alloy. *RSC advances*. **2021**, *11*, 23719-23724. https://doi.org/10.1039/D1RA02073K
2. Wu, L.; Lin, N.; Liu, R.; Yan, K.; Hao, Y.; Wang, W.; Shi, Q.; Yu, Y.; Liu, Z.; Zeng, Q.; Effect of laser surface texturing on sliding wear performance and wear mechanism of CoCrNi and CoCrFeMnNi alloys. *J. Mater. Sci.* **2025**, 1-33. https://doi.org/10.1007/s10853-025-11812-7
3. Zhao, J.-Q.; Tian, H.; Wang, Z.; Wang, X.-J.; Qiao, J.-W.; FCC-to-HCP phase transformation in CoCrNi x medium-entropy alloys. *Acta Metallurgica Sinica (English Letters)*. **2020**, *33*, 1151-1158. https://doi.org/10.1007/s40195-020-01080-6
